# Culture, Self, and Medical Decision Making in Australia and China: A Structural Model Analysis

**DOI:** 10.1177/2381468319871018

**Published:** 2019-09-20

**Authors:** Hankiz Dolan, Dana L. Alden, John M. Friend, Ping Yein Lee, Yew Kong Lee, Chirk Jenn Ng, Khatijah Lim Abdullah, Lyndal Trevena

**Affiliations:** School of Public Health, The University of Sydney, New South Wales, Australia; Ask, Share, Know: Rapid Evidence for General Practice Decision, Centre for Research Excellence, The University of Sydney, New South Wales, Australia; University of Hawai’i, Honolulu, Hawaii; University of Hawai’i, Honolulu, Hawaii; Department of Family Medicine, Faculty of Medicine and Health Sciences, Universiti Putra Malaysia, Selangor, Malaysia; Department of Primary Care Medicine, Faculty of Medicine, University of Malaya, Kuala Lumpur, Malaysia; Department of Primary Care Medicine, Faculty of Medicine, University of Malaya, Kuala Lumpur, Malaysia; Department of Nursing Science, Faculty of Medicine, University of Malaya, Kuala Lumpur, Malaysia; School of Public Health, The University of Sydney, New South Wales, Australia; Ask, Share, Know: Rapid Evidence for General Practice Decision, Centre for Research Excellence, The University of Sydney, New South Wales, Australia

**Keywords:** culture, independence, interdependence, medical decision making, power distance

## Abstract

**Objective.** To explore and compare the influences of individual-level cultural values and personal attitudinal values on the desire for medical information and self-involvement in decision making in Australia and China. **Methods.** A total of 288 and 291 middle-aged adults from Australia and China, respectively, completed an online survey examining cultural and personal values, and their desired level of self-influence on medical decision making. Structural equation modeling was used to test 15 hypotheses relating to the effects of cultural and personal antecedents on the individual desire for influence over medical decision making. **Results.** Similar factors in both Australia and China (total variance explained: Australia 29%; China 35%) predicted desire for medical information, with interdependence (unstandardized path coefficient β_Australia_ = 0.102, *P* = 0.014; β_China_ = 0.215, *P* = 0.001), independence (β_Australia_ = 0.244, *P* < 0.001; β_China_ = 0.123, *P* = 0.037), and health locus of control (β_Australia_ = −0.140, *P* = 0.018; β_China_ = −0.138, *P* = 0.007) being significant and positive predictors. A desire for involvement in decisions was only predicted by power distance, which had an opposite effect of being negative for Australia and positive for China (total variance explained: Australia 11%; China 5%; β_Australia_ = 0.294, *P* < 0.001; China: β_China_ = −0.190, *P* = 0.043). National culture moderated the effect of independence on desire for medical information, which was stronger in Australia than China (*Z* score = 1.687, *P* < 0.05). **Conclusions.** Study results demonstrate that in both countries, desire for medical information can be influenced by individual-level cultural and personal values, suggesting potential benefits of tailoring health communication to personal mindsets to foster informed decision making. The desired level of self-involvement in decision making was relatively independent of other cultural and personal values in both countries, suggesting caution against cultural stereotypes. Study findings also suggest that involvement preferences in decision making should be considered separately from information needs at the clinical encounter.

Shared decision making (SDM) is a process of information exchange between patients and health care providers, working together to make health decisions that are congruent with patient needs, values, beliefs, and goals.^1,2^ It is now a central component of many national health policies and quality standards,^3–6^ but its implementation, particularly within different cultural settings, is potentially challenging. Previous studies have shown that cultural and personal values, sociodemographic characteristics, patient–provider relationships, and types and timing of the decisions can all influence patients’ preferences for involvement in decision making (DM).^3,7–11^ Culture, in particular, has been the focus of a few studies where cultural targeting and tailoring of communication and decision support strategies were suggested to achieve optimal outcomes, such as increased patient satisfaction, adherence, and reduced decisional regret.^7,12,13^ Central to this is the strategy of invoking patients’ deep thinking and choice deliberations by framing information and communication in ways that resonate with their cultural values and individual mindsets.^12,13^ However, this has been challenged by the results of recent randomized controlled trials from the United States, which compared the effects of generic and culturally targeted decision aid materials.^14^ These results suggest that cultural targeting at the group level may not influence the processing of information when individuals are making decisions about options that require slow and deep deliberative thinking.^14^ Rather, the findings suggest that individual-level cultural values/orientations, not the group-level cultural values, influence how individuals process information within medical DM contexts.^14^ Medical DM processes are complex and multidimensional, as are the cultural influences, and more research is needed to examine the interaction between culture, individual self, and medical DM.^15,16^ Findings from such research studies are likely to help inform policy makers, researchers, and practitioners of key cultural and personal attributes of diverse patients that may be most relevant to cultural targeting and tailoring efforts.

Culture is a complex concept and it has many dimensions and layers such as global, national, organizational, group, and individual cultural values.^15,16^ Hofstede’s model of cultural dimensions defines national culture as “the collective programming of the mind distinguishing the members of one group or category of people from others”^17^ and identifies six cultural value dimensions that appear to be distinctive across countries.^17,18^ For example, individualism and collectivism consider the extent to which members of a nation or society are bound together as interdependent agents.^17^ While countries can fall on either the individualistic or collectivist end of the spectrum, individual citizens within a country may differ in their possession of individualistic or collectivist values.^19,20^ Such cultural orientations or values include self-construal, which usually refers to the construction and view of self in relation to others.^20,21^ There are two types of self-construal, interdependent self-construal and independent self-construal, which tend to be more prevalent depending on the national culture’s emphasis on individualism or collectivism.^20,21^ Individuals with greater access to independent self-construal more frequently view themselves as autonomous, distinctive, and characterized by unique internal attributes such as thoughts, feelings, and needs.^21^ Those who possess stronger access to interdependent self-construal, on the other hand, more frequently view themselves as an integral member of a network of social relationships characterized by external attributes such as belonging, roles, and harmony.^21^ An individual can possess both the independent and interdependent self-construal, but one type may be more accessible than the other depending on the situation and context.^19,21,22^ For example, self-construal can be primed or conditioned by national culture.^20^ Individuals within individualist cultures tend to have greater exposure to independent mindsets, whereas people within collectivist cultures tend to be more frequently rewarded for employing interdependent thinking.^20^ Individual self-construal can manifest itself in the form of individual pre-behavioral processes, motivation, and behaviours.^20,23^ For example, “independent individuals” are generally more receptive to messages that emphasize positive personal gain, whereas “interdependent individuals” are more drawn to messages emphasizing loss-avoidance.^24^ In the past, studies from different countries have focused on describing and comparing patterns in patient preferences for self and family involvement within and across cultures.^25–29^ However, only a limited number of studies^7,30,31^ have examined the role that individual-level cultural and personal values play in shaping preferences for involvement in medical decisions and how these relationships are moderated by national-level cultural surroundings.

Alden and colleagues compared the influence of core cultural values on patients’ desired level of medical information and self-involvement in the United States and Japan.^7^ They found that the United States patients’ desire for involvement in the medical DM process was frequently driven by a desire for power-sharing and pursuing personal gain. In Japan, however, the DM process was more likely to be driven by individual values that emphasized the importance of interdependent information exchange.

Extending this work further, we proposed and tested a theoretical model of cultural and personal value predictors of individuals’ desire for involvement in medical DM between a “Western” and “non-Western” national culture—Australia and China. We also looked separately at the desire for health information because people may wish to be well-informed and yet have a preference for less involvement in making a decision.^32^ We explored how individual self-construal and attitudinal values influence patients’ desire for medical information and self-involvement in DM within *and* across two unique cultural settings. The relationship between national-level culture, individual-level cultural values/orientations, and individual desire for information and involvement in medical DM has important implications for the implementation of shared DM policy in different countries and cultures.

## Hypotheses

We modelled three individual-level cultural values (values that are susceptible to national culture conditioning): 1) relational interdependence (RISC), 2) independence (IND), 3) power distance (PD); and one personal attitudinal value, health locus of control (HLC), as antecedents of two parameter values—desire for medical information and desire for self-involvement in DM. Our analysis tested 15 hypotheses (H1–H15), presented in Table 1. Both high interdependent and independent individuals are viewed as active agents in pursuing relational joint goals or personal goals.^33^ Therefore, within the context of medical DM and the doctor–patient dyad, we hypothesized that individuals who place a high value on interdependence or independence should have stronger desire for medical information (H1/H2) and self-involvement in DM (H3/H4). PD is defined by the extent to which individuals accept hierarchical social status and inequity.^18^ It is an individual-level cultural value construct that corresponds with Hofstede’s national culture power distance dimension.^34^ Based on the literature,^7,35,36^ we hypothesized that individuals who value higher PD are more likely to defer DM to authority figures such as health care providers and, therefore, are less likely to desire medical information and involvement (H5/H6). We tested additional hypotheses related to chance (external) HLC, which is characterized by the degree to which individuals view their health as determined by factors that are out of their control.^37^ We hypothesized that HLC would be negatively associated with desire for medical information and self-involvement (H7/H8).^38^

**Table 1:** Hypothesis Testing

	Evidence (Unstandardized Path Coefficients)		Conclusion Supported?
Hypothesis	Australia	China	
H1. Interdependence is positively associated with desire for medical information	.102*	.217**	Yes, in both China and Australia
H2. Independence is positively associated with desire for medical information	.244**	.123*	Yes, in both China and Australia
H3. Interdependence is positively associated with desire for self-involvement in medical decisions	.134	.296	No
H4. Independence is positively associated with desire for self-involvement in medical decisions	.141	−.085	No
H5. Power distance is negatively associated with desire for medical information	.20	.012	No
H6. Power distance is negatively associated with desire for self-involvement in medical decisions	.294**	−.190*	Only in Australia
H7. Chance health locus of control is negatively associated with desire for medical information	−.140*	−.138*	Yes, in both China and Australia
H8. Chance health locus of control is negatively associated with desire for self-involvement in medical decisions	−.081	.168	No
H9. The positive relationship between interdependence and desire for medical information is stronger in China than Australia	*Z* score = −1.447^a^		No
H10. The positive relationship between independence and desire for medical information is stronger in Australia than China	*Z* score = 1.687*^a^		Yes
H11. The positive relationship between interdependence and desire for self-involvement in medical decisions is stronger in China than Australia	*Z* score = −0.887^a^		No
H12. The positive relationship between independence and desire for self-involvement in medical decision is stronger in Australia than China	*Z* score = 1.356^a^		No
H13. The negative relationship between power distance and desire for medical information is stronger in China than in Australia	*Z* score = 1.159^a^		No
H14. The negative relationship between power distance and desire for self-involvement in medical decisions is stronger in China than Australia	*Z* score = 3.939***^a^		No
H15. Desire for medical information indirectly and positively influences desire for self-involvement in both Australia and China	−0.302	−0.047	No

a Critical ratios test for significant differences in path coefficients across groups. Significance indicates moderation by the groups.^39^

* *P* < 0.05. **P < 0.01. ***P < 0.001.

**Figure 1:**
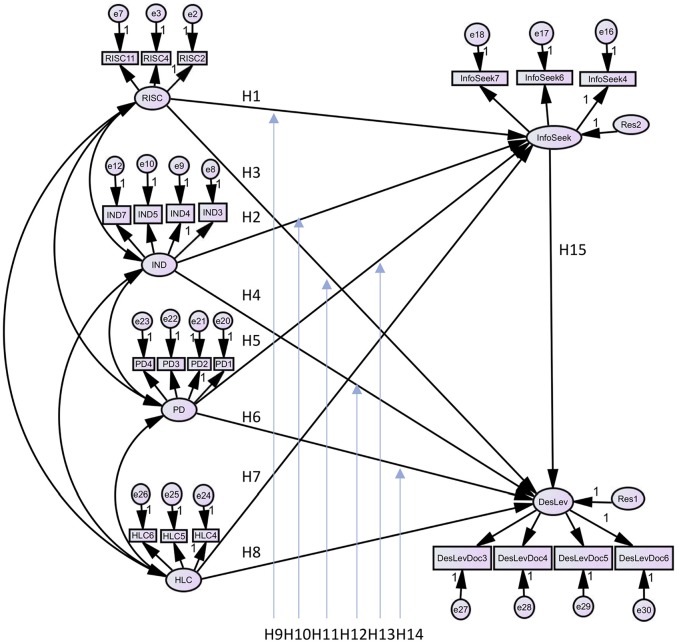
Proposed structural model for both Australia and China. DesLev, desired level of self-involvement; HLC, health locus of control; IND, independence; InfoSeek, desire for medical information; PD, power distance; RISC, relational-interdependence.

In addition to our hypotheses about individual-level predictors of preferences for involvement in DM, we considered the cultural psychology literature on cultural priming and conditioning.^19,21,40^ This suggested that national culture can moderate the effect of individual-level cultural values on desire for DM. Thus, while both the independent and interdependent construal can coexist within an individual, if the national-level cultural context positively rewards or conditions one type of self-view more frequently or stronger than the other, such reinforcement may encourage or normalize subsequent pre-behavioral intentions and behaviors.^19–21^ Australian culture is a highly individualist culture with an index score of 90 on Hofstede’s individualism measure, while Chinese culture is highly collectivist with an index score of 20.^18^ Therefore, we proposed national cultural context moderation hypotheses for interdependence, which we thought would more strongly predict desire for medical information and self-involvement in China (where this type of thinking is more common) than Australia (H9/H11). We hypothesized that having independent values in Australia would be more strongly related to desire for medical information and self-involvement than in China (H10/H12). Similarly, there is a dramatic difference between China and Australia according to Hofstede’s power distance measure (80 v. 36),^17^ with Australians less likely to expect or endorse unequal power distribution. Therefore, we hypothesized that the effect of power distance on the desire for medical information and self-involvement would be stronger and negatively correlated in the Chinese cultural context compared with Australian context (H13/H14). We hypothesized a direct and indirect positive effect from desire for medical information to desire for self-involvement (H15) based on the assumption that patients who want to be more informed will also want to be more involved in their own health care decisions (see Figure 1).

## Methods

### Study design

This study was part of a broader seven-country (China, Australia, Thailand, India, Malaysia, South Korea, and the United States) investigation into culture and its effects on patient–physician DM. A cross-sectional online survey design was used with participants recruited through an online survey company, LightsspeedGMI, which sent e-mail invitations to representative panels of adults aged 30 to 45. This age group was chosen to minimize problems related to familiarity and confidence with the internet. Quota screening maintained equal gender representation in all countries. Participants who voluntarily joined the study received points that could be used to redeem products. A total of 372 and 370 participants in Australia and China, respectively, responded to the survey. Respondents who did not pass the attention test (one item question where participants were instructed to choose a specified answer; 23 in Australia and 12 in China) or took less than 5 minutes to complete the survey (47 in Australia and 55 in China) were removed from the dataset. Furthermore, Tukey’s outlier labelling rule^41^ was applied to remove respondents with extreme responses (14 in Australia and 12 in China). After cleaning the data, missing values (0.12% in Australia and 0.17% in China) were imputed with averages of scale items or single item variables. A total of 288 and 291 responses from Australia and China, respectively, were employed in the final data analysis.

### Measures

The survey instrument consisted of demographic, self-construal (IND, RISC, PD), personal attitudinal (HLC), and contextual items measuring the perceived prevalence of SDM, alongside individual desire for medical information and desire for self and family involvement in DM. In China, this survey was double back-translated to Chinese. Face validity and content validity of the translated scales were assessed and found to be satisfactory. In addition to these validated scales, a 6-item, 5-point Likert-type scale was adapted from similar scenario-based measures published in the past to measure desire for self-involvement in DM across six disease scenarios.^7,26,42–44^ Those scenarios included two minor health problems (common cold and eczema), two moderately serious conditions (diabetes management and asthma), and two very serious conditions (diabetic foot disease and cancer).^26^ A summary of the scales used is presented in Table 2.

**Table 2:** Scale Statistics

SEM Construct	Scale	Scale Source	SEM Items and Scale Type	Cronbach’s α
Desire for medical information	Information-seeking preference scale	Ende et al. (1989)^44^	3 of 8 items 5-point Likert type	Australia .802 China .715
Desire for self-involvement	Six scenario-based scale	Alden et al. (2015)^7^	4 of 6 items 5-point Likert type	Australia .879 China .823
Relational–Interdependence	Relational-interdependent self-construal	Cross et al. (2000)^33^	3 of 11 items 7-point Likert type	Australia .780 China .724
Independence	Independent self-construal scale	Kim et al. (2007)^45^	4 of 14 items 7-point Likert type	Australia .842 China .781
Power distance	Power distance scale	Sharma (2010)^34^	4 of 4 items 7-point Likert type	Australia .848 China .805
Health locus of control	Chance health locus of control scale	Wallston et al. (1978)^37^	3 of 6 items 4-point Linkert type	Australia .743 China .781

SEM, structural equation modeling.

### Data Analysis

As the first step in model validation, exploratory factor analysis was carried out with maximum likelihood estimation and Promax rotation. Items that had loadings close to or above 0.500 on a single factor in both countries were retained for the next step of confirmatory factor analysis (CFA) to ensure convergent validity.^46^ During CFA, bootstrapping (1000 bootstrap samples) was used in both countries due to violations of multivariate normality.^47^ Items with poor loadings (below 0.5) or higher modification indices for model improvement were dropped. Final items for each structural equation model (SEM) construct and summary statistics are presented in Table 2.

For the self-involvement construct, only items related to the moderate and serious scenarios were entered in the final model. The modified measurement model fit indices for both China and Australia are shown in Table 3. In both countries, the measurement model convergent validity was investigated using the average variance extracted (AVE) estimates.^46^ The master validity plugin tool developed by Gaskin and Lim was used to extract relevant results from Amos.^48^ In the Australian model, except for HLC (0.491), all other AVE estimates were above 0.50. In the Chinese model, AVE estimates for RISC (0.472), IND (0.494), and medical information desire (0.471) were slightly below recommended 0.50.^46^ Fornell and Larcker^49^ suggest that convergent validity can still be treated as adequate if Composite Reliability (CR) alone is satisfactory. In our study, all CRs were above 0.7, meeting the minimum satisfactory threshold.^46,49^ In addition, all AVE estimates were larger than maximum shared variance and the square roots of AVE estimates were greater than interconstruct correlations, indicating satisfactory discriminant validity in both countries^46^ (see Table 4). The variance inflation factors for all independent variables were below 2, indicating that multicollinearity was not a concern in either country.^46^ Common method bias was examined by controlling for the effects of a single unmeasured latent factor.^50^ The proportion of change in variance after adding a common latent factor was 17% in Australia and 16% in China. Both were lower than the common threshold of 50%, and therefore, less likely to have significant effects on the regression weight outcomes.^50,51^

**Table 3:** Model Goodness of Fit Indices

	χ^2^	DF	CMIN/DF	CFI	TLI	SRMR	RMSEA	PCLOSE
Recommended^46^			<3	>0.92	>0.92	<0.08	<0.07	>0.05
Chinese measurement model	307.78	174	1.769	0.933	0.919	0.054	0.051	0.386
Australian measurement model	327.514	174	1.882	0.924	0.930	0.053	0.055	0.162
Unconstrained combined measurement model	635.297	348	1.826	0.938	0.926	0.054	0.038	1.000
Fully constrained combined measurement model	659.151	369	1.786	0.938	0.929	0.056	0.037	1.000
Structural model	635.297	348	1.826	0.938	0.926	0.054	0.038	1.000

CFI, comparative fit index; CMIN, relative chi-square; DF, degrees of freedom; RMSEA, root mean square error of approximation; SRMR, standardized root mean square residual; TLI, Tucker–Lewis index.

**Table 4:** Model Validity Measures^a^

	CR	AVE	MSV	1	2	3	4	5	6
Australia									
1. Relational-interdependence	0.782	0.544	0.092	**0.738**					
2. Independence	0.848	0.583	0.234	0.303	**0.764**				
3. Desire for medical information	0.806	0.583	0.234	0.295	−0.077	**0.763**			
4. Power distance	0.850	0.588	0.169	0.182	0.006	0.411	**0.767**		
5. Health locus of control	0.743	0.491	0.169	0.141	−0.034	0.151	0.284	**0.701**	
6. Desire for self-involvement	0.901	0.696	0.081	0.484	−0.141	0.104	−0.055	0.060	**0.834**
China									
1. Relational-interdependence	0.728	0.472	0.394	**0.687**					
2. Independence	0.793	0.494	0.394	0.627	**0.703**				
3. Desire for medical information	0.726	0.471	0.264	0.514	0.010	**0.686**			
4. Power distance	0.806	0.511	0.169	0.194	−0.094	0.411	**0.715**		
5. Health locus of control	0.781	0.545	0.169	0.050	0.010	0.155	−0.078	**0.738**	
6. Desire for self-involvement	0.827	0.546	0.024	0.481	−0.213	0.072	0.040	0.057	**0.739**

AVE, average variance extracted; CR, composite reliability; MSV, maximum shared variance.

a Figures in bold: square root of AVE.

In order to validate comparisons of structural path coefficients across countries, configural and metric invariance tests were performed. The model fit was acceptable when both countries loaded together in a combined model, showing adequate configural invariance. When combined model factor loadings were constrained to be equal across groups, the chi-square difference test between the fully constrained model and the unconstrained model was not statistically significant (*P* = 0.298), meaning full metric invariance was supported. This level of invariance allowed comparisons of unstandardized path coefficients across countries but not means.^52^

## Results

### Sample Descriptions

The age and gender distribution across the Chinese and Australian samples were similar due to quota screening during recruitment. Samples differed statistically on the distribution of income, education, occupation, and marital status (see Table 5). Compared to their respective national population distributions, both were skewed toward middle and high income, high educational attainment, and professionals and administrative positions.^26^ This similarity in sample skew between the two countries is likely to reduce the possibility of sociodemographic confounding effects on the results.^26^ In both countries, the average desire for medical information was toward the high end of the scale, while the desire for self-involvement was closer to the midpoint.

**Table 5:** Characteristics of the Sample^a^

	Australia, *N* (%)	China, *N* (%)
Age, mean (SD)	37.31 (4.328)	36.4 (4.0)
Gender		
Male	145 (50.3)	147 (50.5)
Female	143 (49.7)	144 (49.5)
Marital status		
Single	72 (25.0)	17 (5.8)
Married*	157 (54.5)	268 (92.1)
Living with partner	41 (14.2)	1 (0.3)
Divorced/separated	17 (5.9)	5 (1.7)
Widower/widow	1 (0.3)	0
Education		
Junior high school (K–8th grade)		0
Part senior high school (some high school)	3 (1.0)	1 (0.3)
High school (high school graduate or GED)	23 (8.0)	2 (0.7)
Junior college degree (some college or 2-year degree)	83 (28.8)	56 (19.2)
Bachelor degree (4-year college degree)*	119 (41.3)	209 (71.8)
Postgraduate degree	60 (20.8)	23 (7.9)
Income level		
Less than RMB 12,500 (less than $25,000)*	14 (4.9)	54 (18.6)
12,500–25,500 ($25000–50,000)	41 (14.2)	64 (22.0)
25,501–38,000 ($50,001–75,000)	66 (22.9)	60 (20.6)
38,001–51,000 ($75,001–100,000)	71 (24.7)	33 (11.3)
51,000–63,000 ($101,000–$125,000)	42 (14.6)	25 (8.6)
>63,000 (more than $125,000)	54 (18.8)	55 (18.9)
Overall health		
Very poor	3 (1.0)	0
Poor	9 (3.1)	13 (4.5)
Fair	68 (23.6)	95 (32.6)
Good	121 (42.0)	95 (32.6)
Very good	64 (22.2)	83 (28.5)
Excellent	23 (8.0)	5 (1.7)
Occupation		
Housewife	42 (14.6)	8 (2.7)
Professional (medical, lawyer, teacher, etc.)	78 (27.1)	51 (17.5)
Private or public sector managerial, executive*	45 (15.6)	142 (48.8)
Private or public sector administrative, clerical	58 (20.1)	48 (16.5)
Skilled craft, trade, or service provider	32 (11.1)	33 (11.3)
Semiskilled worker		6 (2.1)
Military	1 (0.3)	0
Student	10 (3.5)	0
Other	22 (7.6)	3 (1.0)
SEM Construct (Scale Score Range)	Mean Score (Range)	
Desire for medical information (1–5)	4.54	4.48
Desire for self-involvement (1–5)	3.29	3.09
Relational interdependence (1–7)	5.32	5.63
Independence (1–7)	5.99	5.78
Power distance (1–7)	4.31	4.46
HLC (1–4)	2.36	2.07

*N*, total number; SD, standard deviation.

a Significance in difference in proportions or mean score: *P < 0.05, **P < 0.01, ***P < 0.001.

### Structural Model Validation

As in the CFA analysis, bootstrapping (1000 samples) was used to estimate the structural model. The model fit indices were acceptable (see Table 3), allowing the analysis of path coefficients. Overall, in Australia, 29% of the variance in the desire for medical information was explained by predictor variables, and in China, the figure was 35%. As for desire for self-involvement, the predictor variables accounted for 11% and 5% of the total variance, respectively.

**Figure 2:**
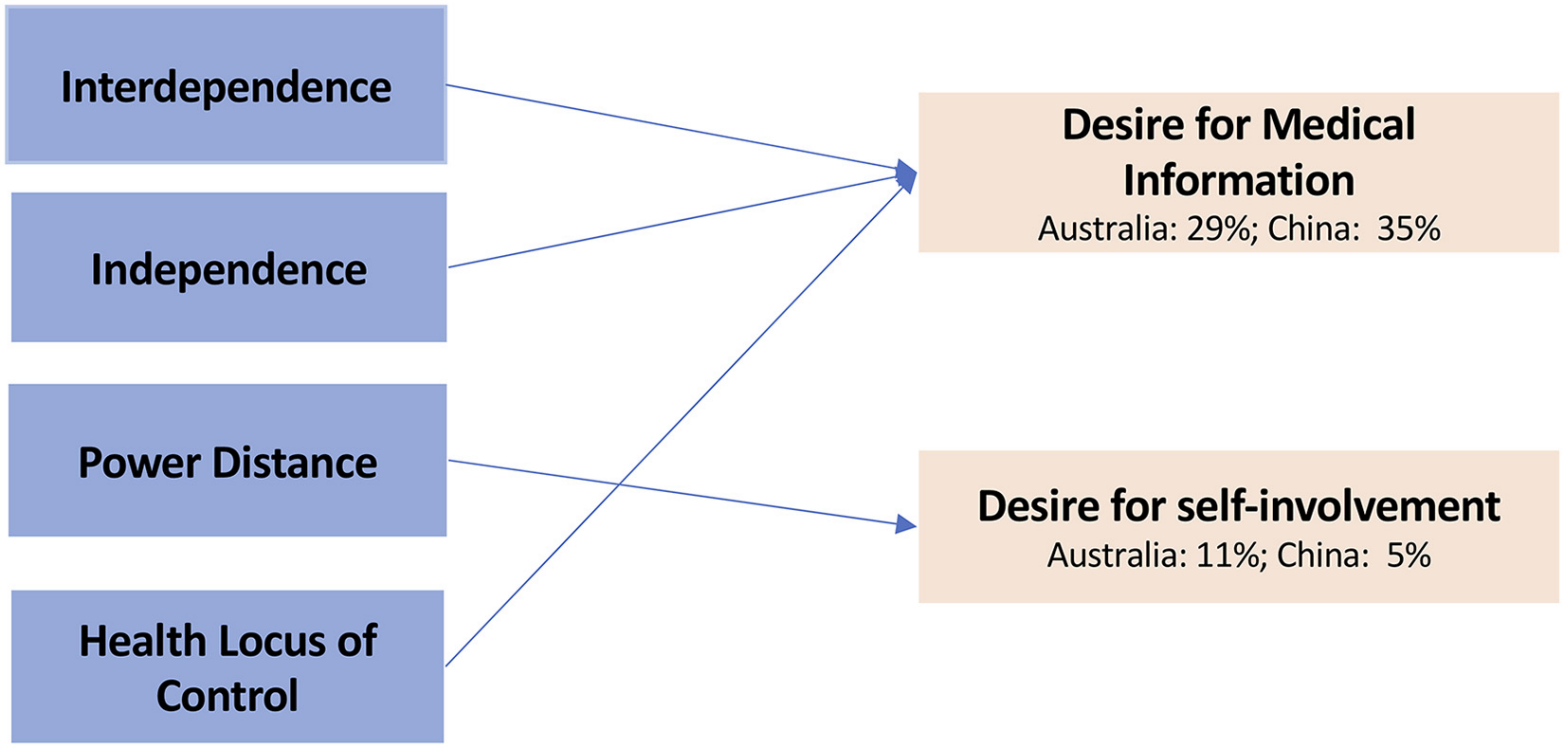
Structural equation model: Australia and China (significant paths only, *P* < 0.05). Percentages: Total variance explained.

In both Australia and China, hypotheses were supported regarding the positive influence of the individual-level cultural values (interdependence and independence) on the desire for medical information (H1 and H2) and negative influence of the personal value of HLC on the desire for medical information (H7) (see Figure 2 and Table 1). Contrary to H5, power distance had no effect on desire for medical information in either country. In addition, paths from interdependence (H3), independence (H4), and health locus of control (H8) to desire for self-involvement were not significant. However, while the hypothesized negative association of power distance and desire for self-involvement (H6) was supported in Australia, the opposite was observed in China, where power distance positively influenced the level of desire for self-involvement. Desire for medical information was not related to desire for self-involvement in either country (H15).

Furthermore, our hypothesis of national cultural context moderation of antecedent effects on outcomes was supported for the path from independence to desire for medical information (H10), which was positive and significant in both countries. A test of the strength of the unstandardized path coefficient revealed that this relationship was significantly stronger in Australia versus China (*P* ≤ 0.05, one-tail test). Finally, our initial hypothesis of power distance having a negative influence on the desired level of self-involvement (H6) was supported in Australia, but rejected in China, where it had the opposite effect.

## Discussion

This study investigated the impact of cultural and personal factors on desire for medical information and desire for self-involvement in medical decisions in Australia and China. In both countries, the desire for medical information was found to be high, driven by individual mindsets of benefiting each other through knowledge sharing (interdependence) and by pursuing individual interests through obtaining information (independence). In both cultures, having higher levels of chance health locus of control undermined individuals’ desire for medical information, possibly stemming from beliefs that health is controlled by luck or fate.^38^ On the other hand, individual-level cultural and personal factors (IND, RISC, and HLC) were *not* predictors of preferences for self-involvement in medical decisions.

Interestingly, the expected negative association between power distance (value placed on social hierarchy) and desire for self-involvement was found only in Australia, where individuals having a greater respect for authority may be more likely to defer DM to their doctor as a person of authority. However, we found the opposite effect of individual power distance values in China, where individuals with a higher respect for authority were less likely to defer DM to their doctor. The unexpected contradictory effect of power distance cultural scores on self-involvement in DM within the Chinese sample was inconsistent with prior research findings.^7,53–56^ Empirical evidence suggests that people who place greater value on social hierarchy and status are less willing to participate in a DM process involving someone who is perceived as powerful, authoritative, and superior.^18,53,56,57^ The imbalance of power within the doctor–patient dyad is often more weighted toward doctors because doctors are perceived by patients as someone who has the professional expertise to cure the disease whereas they cannot do it themselves.^29,53,54,58^ This caused us to question the power dynamics within the doctor–patient relationship in Chinese culture.

A recent report by Zhou and colleagues^59^ describes issues arising from the changing doctor–patient relationship in China. In the past, heavily influenced by traditional Chinese culture, doctors were highly respected and regarded as “white angels” who saved lives.^59^ However, since 1978, market-oriented health sector reforms and rapid increases in health care costs have been associated with a decline in trust between doctors and patients.^59^ High expectations from patients fueled by rising medical costs, and negative media reports about the conduct of doctors and hospitals, have contributed to this issue.^59^ The relationship between patients and doctors in China has been described as adversarial, with health care being seen as a commodity.^60,61^ This is quite a contrast to the health care model in Australia, where the universal health insurance scheme, Medicare, has been in place since 1984.^62^ For all eligible Australian citizens and residents, Medicare covers all medical costs for in-hospital and ambulatory care in public hospitals and all or up to 85% of the scheduled fees set up by the Medicare for consultations with primary care specialists.^62^ There is generally a high level of trust toward health care professionals in Australia, with nurses and doctors being rated as the most highly regarded professions in terms of ethics and honesty.^63^ Therefore, it could be that in China, the more patients value social hierarchy, the more they would expect to be involved in their health care decisions as their doctors are regarded as less superior/trustworthy service providers. While the adversarial relationship and trust issues between health care providers and patients may have contributed to our unexpected result in China, given that the final model explained only 5% of the variance in the desire for involvement in China, future research is needed to further examine this relationship and its influencing factors.

Our results also highlight that a desire for medical information did not predict a desire for self-involvement in either country. There are mixed findings in the literature in terms of the relationship between these two indicators of patient preferences for autonomy.^44^ For example, in a study by Alden and colleagues, a strong association between these two indicators was found in Japan but not in the United States.^7^ Similarly, other studies have found that patients who were active in information seeking did not necessarily prefer to be actively involved in DM or vice versa.^64,65^ Our results further add to evidence suggesting that preference for participation in decisions is influenced by complex and intertwined individual and contextual factors, of which preference for information is just one potential indicator.^9^ Overall, these findings suggest that involvement preferences in DM should be considered separately from information needs at the clinical encounter.

We also tested the national-level cultural moderation effect on the relationships between the individual-level cultural values and a desire for medical information. We found that, as expected, independence (individualism) was a stronger predictor of desire for information in Australia, but surprisingly, interdependence (collectivism) was *not* a stronger predictor of desire for information in China, breaking down some stereotypes. Cultural moderation did not appear to be significant for desire for self-involvement with the exception of power distance as mentioned earlier. Our study further confirms that desired level of self-involvement in DM is relatively independent of other cultural and personal attitudinal factors and that health care providers should equally provide patients with opportunities to deliberate and act on their preferred role in DM. These findings are consistent with SDM paradigms that advocate for the right to high-quality information for all patients, but a respect for individual variability in terms of preference for level of involvement when making health decisions.^32^

### Future Research and Limitations

Our findings have several important implications for future research and practices that aim to encourage SDM. There are two aspects to the patient desire for involvement in DM: desire for medical information (being informed) and desired level of involvement in making the final decision.^44^ First, since both independent and interdependent values can predict desire for medical information, consideration should be given to tailoring health information and communication strategies to individual cultural and personal values in order to increase receptiveness.^7,12,14,24^ Recent study findings from four randomized controlled trials in the United Stated found that personal level of interdependent values are predictive of decision aid materials’ impact on decision preparedness, such as knowledge, decision conflict, and empowerment.^14^ Therefore, individuals could benefit from being provided with information that resonates with their own self-construal and values to facilitate “internal deliberations.”^12,14^ For example, in order to invoke a patient’s desire for medical information, health information or dialogue could incorporate cues for mutual benefit (interdependence) or personal gain (independence), depending on the patient’s dominant self-construal. The move toward developing the skills of SDM, such as values elicitation and clarification, within both countries is likely to assist this process. Second, given the negative impact of health locus of control on desire for health information in both countries, the need for building mutual trust for opening discussion on evidence-based information seems especially important with patients who attribute their health to chance, fate, or luck. Third, individual values for power distance (respect for authority) were not predictive of desire for information in either country. This has important implications for practitioners and policy makers in ensuring equitable and widespread sharing of information with patients across health care settings. Health care practitioners should provide opportunities for informed DM regardless of patients’ cultural backgrounds and attitudes toward health care practitioners’ authority/power. Finally, since social status/power distance predicted patients’ desire for self-involvement in making the final decision in both Australia and China, future interventions could benefit from bridging the power gap and promoting equity and trust between patients and providers. In Australia, patient empowerment interventions could benefit from advocating patients’ rights to be respected, informed, and included in their health care decisions as set out in the Australian Charter of Healthcare Rights.^66^ In China, caution should be exercised when designing such interventions so as not to inadvertently worsen patient–doctor relationship and trust issues. A stronger emphasis on mutual respect, trust, and two-way communication seems especially imperative in China to close the patient–provider power gap. Future studies in China are needed with samples that are more representative in order to further examine patient–doctor power relationships and their effects on DM.

More important, given that desire for self-involvement was found to be relatively independent of all the other factors that were included in the model, we recommend that physicians avoid overgeneralization based on cultural stereotypes and assess each patient on an individual basis.^26^ As discussed earlier in reference to Hofstede’s work,^18^ Australians as a whole tend to have more individualist values, while Chinese tend to be more collectivist. For these reasons, Australian individuals are often presumed to value independence more than individuals with Chinese cultural backgrounds, who are presumed to value interdependence more. However, our study finds that neither interdependent nor independent mindsets predict a desire to be involved in making medical decisions. Furthermore, power distance was associated with a reduced desire for involvement in Australia but increased desire in China. Therefore, we recommend that in the absence of specific assessment for a preferred role in DM in clinical situations, health care providers avoid assumptions based on patients’ cultural background or home-country and provide each patient with opportunities to be involved in the DM process.

This study has several limitations. First, as an online cross-sectional study, fewer representatives from disadvantaged groups may have participated, and as a result, participation bias is a possibility.^67^ Participants were generally highly educated, had good incomes, and held professional or administrative positions. Therefore, our sample was not representative of the overall populations in Australia or China. However, we aimed to test a theoretical model to see if certain cultural and personal values could predict desire for medical information and desire for self-involvement, rather than to reach conclusions on the DM preference or behaviors of whole population groups. Therefore, our study demonstrated that those relationships between proposed constructs in our model “can” happen, even if they were among certain population groups.^68^ Future research should explore whether these findings are valid among different age groups or people with diverse socioeconomic backgrounds. Second, we used hypothetical scenarios to assess desire for self-involvement in DM and only four moderate and serious scenario items were entered into the final model. These findings may not necessarily reflect the contexts in which patients are faced with real choices. Furthermore, patient preferences for involvement in DM could vary across different disease conditions and timing of the disease and caution should be given to overgeneralization.

Despite such limitations, our current study demonstrates the complexity of the processes behind patients’ desire to influence their own medical DM. We have shown that individual level cultural values and attitudes can predict one’s desire to influence medical decisions to some degree. However, these predictors alone do not explain the full variance in patient’s desire for medical information and involvement in DM. Thus, while attention to these individual-level cultural and attitudinal values could benefit efforts to foster informed and shared DM, overgeneralization and stereotypes based on cultural backgrounds should be avoided.
